# Screening for primary aldosteronism in 1,181 Swedish primary care patients with hypertension

**DOI:** 10.3389/fendo.2025.1555572

**Published:** 2025-04-14

**Authors:** Nikita Makhnov, Jakob Skov, Tobias Åkerström, Fredrik Axling, Daniel Andernord, Mikael Bergenheim, Mauritz Waldén, Per Hellman

**Affiliations:** ^1^ Department of Surgical Sciences, Uppsala University, Uppsala, Sweden; ^2^ Department of Surgery, Karlstad Central Hospital, Karlstad, Sweden; ^3^ Center for Clinical Research and Education, Karlstad, Sweden; ^4^ Department of Medicine, Karlstad Central Hospital, Karlstad, Sweden; ^5^ Department of Molecular Medicine and Surgery, Karolinska Institutet, Stockholm, Sweden; ^6^ Department of Surgery, Uppsala University Hospital, Uppsala, Sweden; ^7^ Department of Health Sciences, Karlstad University, Karlstad, Sweden

**Keywords:** primary aldosteronism, screening, hypertension, outpatients, aldosterone, renin, therapeutics

## Abstract

**Objective:**

Primary aldosteronism (PA) is a common cause of hypertension. It entails elevated morbidity and mortality that do not sufficiently improve with conventional antihypertensive therapy. Screening for PA by plasma aldosterone–renin ratio (ARR) enables discovery and specific treatment of affected patients. By screening primary care patients with hypertension and evaluating them further according to the Endocrine Society guidelines, we aimed to assess the prevalence of PA, the factors affecting biochemical diagnostics, and the outcome of lateralization studies and of specific treatment of the discovered PA cases.

**Design, patients, and methods:**

Prospective evaluation of screening for PA was conducted in 1,181 patients. Screening by ARR was performed under current therapy, but without mineralocorticoid receptor antagonists (MRA), under normokalemia, and confirmed by the intravenous saline suppression test, SST#1. Those with results in a defined gray zone underwent therapy adjustment and then completed SST#2. Plasma aldosterone and ARR were compared under different stages of the diagnostic process. All patients with PA were offered adrenal venous sampling, or, in certain cases, adrenocortical-specific positron emission tomography. Lateralizing cases were offered laparoscopic adrenalectomy. Patients with bilateral disease were treated with MRA. Treatment results were assessed after a minimum of 6 months.

**Results:**

A total of 53 discovered cases of (mostly mild) PA corresponded to its prevalence of 4.5%. Initial seated ARR was higher than recumbent ARR before SST#1. At SST#2, initial ARR and final aldosterone were higher than at SST#1. Localizing studies (accepted by 45 patients) found 14 lateralized cases. Of the 11 operated cases, 4 had aldosterone-producing adenoma, and the remainder had micro- and macronodular histopathology. A total of 31 patients had bilateral PA. Both surgical and conservative treatments were well tolerated and led to improved blood pressure and higher renin, indicating risk amelioration.

**Conclusions:**

PA is prevalent among primary care patients with hypertension and can be screened for under current antihypertensive therapy. Aldosterone-producing adenoma was rare in this cohort. The study results support active screening of primary care patients with hypertension for PA in order to offer appropriate treatment options.

## 1 Introduction

Primary aldosteronism (PA), manifested by excessive autonomous production of aldosterone in the adrenal cortex, is the most common cause of secondary hypertension (1). Arterial hypertension globally affects over 30% of adults 30–79 years old (2), and a considerable part of these patients have documented PA (3–5). In particular, high prevalence of PA is shown in resistant or refractory hypertension (6). Moreover, PA comprises a continuum of subclinical to clinical conditions where the risk of cardiovascular, metabolic, and renal disorders rises along with the degree of hyperaldosteronism—unrelated to blood pressure control *per se* (7–11). Accumulating evidence supports the existence of subclinical forms of PA—preceding the development of hypertension (7–9, 12). The public health aspect of PA is underlined by the fact that, compared with primary hypertension, PA considerably worsens cardiovascular morbidity and mortality, which cannot be effectively controlled by traditional antihypertensive treatment (13–15), at the same time as specific surgical and medical treatment strategies exist (16–19). To be able to offer the patients the best possible treatment, it is necessary to evaluate if the pathologic aldosterone production takes place in only one or in both adrenals, which requires adrenal vein sampling (AVS), and lately may also be evaluated with the introduction of adrenocortical positron emission tomography/computed tomography (PET/CT, 20, 21). PA often presents no pathognomonic symptoms, which is why biochemical screening—most often by aldosterone–renin ratio (ARR)—has hitherto been necessary for the identification of suspicious cases, requiring further confirmatory diagnostic workup (20). In some instances of much elevated ARR combined with hypokalemia, the confirmatory testing might be superfluous (20, 22).

The Endocrine Society guidelines (20) describe a PA prevalence of approximately 5%–13% among patients with hypertension. Previous studies in Sweden involving small numbers of patients showed a prevalence of PA of up to 5.5% in newly diagnosed hypertensives and 8.5% in known hypertensives in a primary care setting (23, 24). The latest meta-analysis of the PA prevalence was hard to interpret, mainly due to the heterogeneity of the various included study designs, diagnostic methods, and criteria (5, 25–27). There exists an intimate connection between assessed prevalence rates of PA and its diagnostic thresholds, which are strikingly different at different centers (28), and with a tendency towards higher prevalence rates in more recent studies (15). At this moment, a PA prevalence of at least 10% of all individuals with hypertension and at least 20% in those with treatment resistant hypertension (15, 29) seems probable, while the prevalence in cohorts considered to have low risk for PA is more uncertain (8). Today, less than 2% of patients with risk of PA are screened within routine clinical practice (15).

Pathophysiologic research into the cellular changes in the affected adrenals in PA has recently resulted in the HISTALDO consensus on histological forms of the disease (30). Introduction of immunohistochemical (IHC) analysis of the enzyme aldosterone synthase, encoded by the gene *CYP11B2*, has made it possible to discern aldosterone production in removed adrenal specimens, thus defining previously unseen aldosterone-producing micronodules [APMs, earlier named aldosterone-producing cell clusters (APCCs)], as well as aldosterone-producing nodules (APNs) and aldosterone-producing adenomas (APAs). Contrary to previous understanding, it now seems that the majority of unilateral PA is represented by such nodular forms and to a lesser extent by APAs, whereas diffuse CYP11B2-positive hyperplasia is unusual (31, 32). Unilateral PA is definitely best treated by surgery (33).

Using ARR, we have performed a large screening in primary care individuals with hypertension and evaluated them further according to the Endocrine Society guidelines. The aims were to estimate the prevalence of PA in an unselected cohort of primary care patients with hypertension in Sweden; to subclassify the found patients; to describe the practical complexity of the guidelines-recommended approach; and to report the results of diagnostic evaluation, as well as the outcome of offered treatment.

## 2 Materials and methods

### 2.1 Study population and recruitment

**Figure 1:**
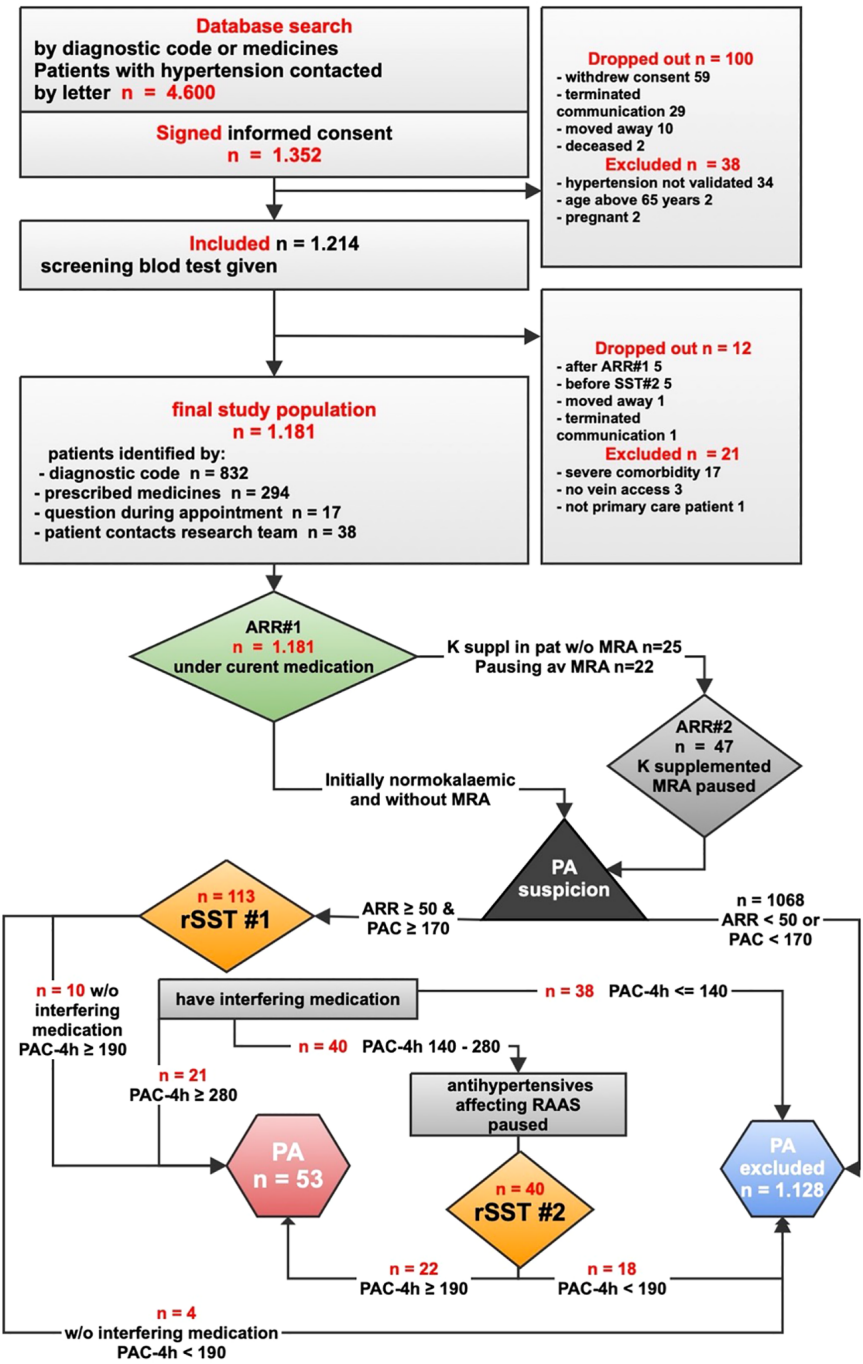
Study flowchart 1—screening for and diagnosing primary aldosteronism. ARR, aldosterone–renin ratio (pmol/mIU); MRA, mineralocorticoid receptor antagonists; K, potassium; K-suppl in pat w/o MRA, potassium supplementation in patients without MRA; PAC, plasma aldosterone concentration (pmol/L); rSST, recumbent saline suppression test; PAC4h, plasma aldosterone concentration at the end of rSST; RAAS, renin–angiotensin–aldosterone system; PA, primary aldosteronism.

**Figure 2:**
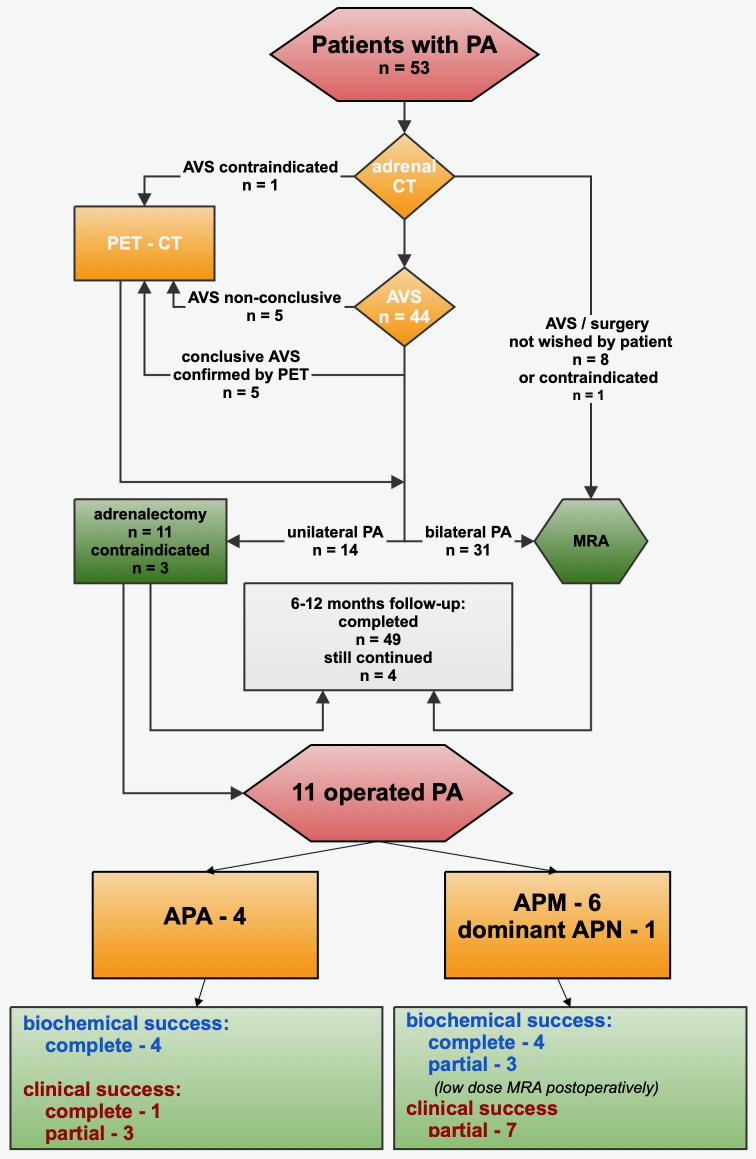
Workup and treatment of 53 cases of primary aldosteronism; pathological, biochemical, and clinical outcomes of operated cases. PA, primary aldosteronism; CT, computed tomography; dexa-cortisol, dexamethasone suppressed cortisol; AVS, adrenal venous sampling; PET-CT, positron emission tomography with computed tomography; MRA, mineralocorticoid receptor antagonists.

**Figures 1** and **2** summarize the study protocol. A regional healthcare database was used to access postal addresses of primary care patients who had a registered diagnosis code for hypertension and/or were taking antihypertensive medicines. The search was done for patients 18–65 years old, pertaining to the participating outpatient clinics located in and around Karlstad (the central city of the Swedish region of Värmland). The patients were then contacted by letter containing study information, a health questionnaire, and a written consent form that, if signed, gave the study personnel access to the relevant medical chart data. Information posters were displayed at the participating primary care centers, giving patients with hypertension a possibility to contact the study personnel and receive the same letter. For individuals who consented to participation, diagnosis of arterial hypertension was validated through medical charts by either presence of a diagnosis code for hypertension or at least three instances of blood pressure ≥ 140/90 mmHg at rest. Inclusion and exclusion criteria are described in **Table 1**. Self-reported data, as the age at which hypertension was discovered, cardiovascular diseases, and current medication, were validated through the patients’ medical charts. Tobacco and licorice use were self-reported.

**Table 1:** Inclusion and exclusion criteria.

Inclusion criteria:		
Validated diagnosis of arterial hypertension		
Age		18–65 years
Possession of Swedish personal number		Permits elective medical care
Exclusion criteria:		
Pregnancy		
Patients followed for PA or hypertension within specialized centers and not within primary care		
Conditions rendering diagnostic protocol (rSST#1^a^, pausing of MRA or other medicines before ARR#2^b^ or rSST#2^c^) a high-risk procedure (as described on the right):^d^	Hypertension with blood pressure levels > 180/110 in spite of rigorous within-study efforts to optimize treatment	
	Chronic heart failure, New York Heart Association stage > 2	
	Severe respiratory, hepatic, or renal failure	

a rSST#1, recumbent saline suppression test number 1 (see study protocol in **Figure 1**).

b ARR#2, aldosterone–renin ratio number 2 (see **Figure 1**).

c rSST#2, recumbent saline suppression test number 2 (see **Figure 1**).

d Patients who were deemed not fit for rSST or for pausing of medication when necessary were excluded from further study participation and advised to be initiated/continued on a clinically adjusted dose of mineralocorticoid receptor antagonists (MRA) through their primary care physician.

Calculations assuming a prevalence of 5% of PA within the screened population, a type 1 error of maximum 0.05, and a power of 80% resulted in a necessary number of screened individuals to be 1,168. Altogether, 1,214 patients who signed the informed consent and actually gave the first screening blood test were included in the study, and 1,181 of them completed the study. Two of the patients who signed the informed consent had already been diagnosed with PA before through routine clinical investigation corresponding to the study protocol. They were included but not reinvestigated. Inclusion in the study took place between April 2017 and June 2022, with a remarkable delay of the screening and diagnostic procedures during the COVID-19 pandemic.

### 2.2 Overview of the screening and diagnostic protocol

In an attempt to allow greater sensitivity and simplicity in screening, the initial ARR (ARR#1) was taken in all included subjects and under current medication. Otherwise, the screening, confirmation of the suspected PA cases (requiring adjustment of medication in some patients), and evaluation for lateralization of the disease in potential surgical candidates were performed according to the Endocrine Society guidelines. Calculation of ARR was done by dividing the plasma aldosterone concentration (PAC) by direct renin concentration (DRC). Even later in the study, renin was assessed as DRC. When the DRC was below the lower limit of 1.6 mIU/L reported by the laboratory, then ARR was calculated using a DRC value of 1.6 mIU/L.

#### 2.2.1 Screening procedures

Before screening, the patients were advised a 2-week run-in period with liberal salt intake and abstinence from licorice and chewing tobacco. Blood samples were taken at 8–10 a.m., ideally 2 h after awakening and being in upright position. A period of seated rest of 5–15 min was followed by measuring blood pressure and then obtaining blood samples: plasma sodium, potassium, creatinine, and ARR (denoted ARR#1, thus taken under current antihypertensive medication and, at this moment, regardless of the current MRA use or potassium level). See **Figure 1** for more details.

Normokalemic patients without MRA, with PAC < 170 pmol/L, or with ARR < 50 pmol/mIU were considered not to have PA and thus finished their study participation. In case of PAC ≥ 170 and ARR ≥ 50, PA was suspected.

Individuals with plasma potassium <3.5 mmol/L received oral potassium substitution (continued throughout the diagnostic assessment) and were evaluated later under normokalemia. If initial PAC (PAC#1) or ARR#1 at the time of hypokalemia was below the diagnostic threshold, another ARR (ARR#2) was taken under normokalemia.

All patients taking MRA were advised a period of MRA discontinuation, which was effectuated under close clinical supervision and control of plasma potassium. If successfully paused, MRA was discontinued during the remaining diagnostic procedures. At least 6 weeks after MRA discontinuation, ARR#2 was taken. An ARR#2 ≥ 50 pmol/mIU in combination with PAC#2 ≥ 170 pmol/L motivated confirmation testing. Patients with ARR ≥ 50 pmol/mIU and PAC < 170 pmol/L were considered as not being eligible for confirmation testing.

#### 2.2.2 Confirmation of PA

Confirmation of PA was performed using the recumbent intravenous saline suppression test (rSST). At the time of initiating the study, rSST was the gold standard, which today has changed to seated SST. The effort was made to maintain normokalemia prior to rSST and not to introduce new antihypertensive preparations with known effect on the renin–angiotensin–aldosterone system (RAAS).

The rSST was initiated at 8–10 a.m. Infusion of 2 L of physiologic sodium chloride solution was given during 4 h under repetitive control of blood pressure, pulse, and wellbeing. Thirteen patients whose blood pressure was above 165/105 received doses of doxazosin, verapamil, or amlodipine. Most patients (*n* = 106 of 113) needed no additional medication under rSST#1, and no patient experienced any discomfort.

The results of the rSST#1 were interpreted according to the guidelines (20) (**Figure 1**). If final aldosterone (PAC-4h) was ≤140 pmol/L, PA was deemed not present, and the study participation was discontinued. If PAC-4h was ≥280 pmol/L, the diagnosis of PA was confirmed. Aldosterone within the “gray zone” of 140–280 pmol/L was denoted “possible PA”.

The patients with “possible PA” who took no antihypertensive preparations besides long-acting calcium blockers and/or doxazosin were not diagnosed with PA if PAC-4h after rSST#1 was below 190 pmol/L or diagnosed with PA if PAC-4h was ≥190 pmol/L, as suggested by the guidelines (20).

Patients with “possible PA” taking any other antihypertensive drugs were advised to temporarily and gradually discontinue those and substitute those with long-acting calcium blockers and/or doxazosin, under close supervision of a research physician. Six weeks after complete substitution as above, the patients underwent rSST#2. In 12 cases, the complete discontinuation and substitution was deemed unsafe, and rSST#2 was performed under a maximal clinically acceptable change of medication. In some cases, rSST#2 was done 2–4 weeks after maximally optimized medication due to reduced clinical tolerance of the medication change. All rSST#2 procedures were well tolerated, with no need for extra hypotensive medication.

During rSST#2, according to the guidelines (20), a PAC-4h of ≥ 190 pmol/L was considered as proof of PA. If PAC-4h was <190 pmol/L, then the patients were not diagnosed with PA and their study participation was terminated. One patient who could not discontinue nonsteroidal anti-inflammatory drugs (NSAIDs) had clinically probable PA, which was confirmed in spite of a PAC-4h of 180 pmol/L after rSST#2. Good subsequent blood pressure response to MRA served as an additional proof of PA in that case.

Our intention was to assure normokalemia before proceeding to both rSST#1 and rSST#2. Nevertheless, in some cases, hypokalemia occurred anyway, but if PAC-4h of rSST#1 was below 140 pmol/L, then rSST#2 was completed after optimized potassium substitution. Subjects not diagnosed with PA were referred back to their primary care physician.

#### 2.2.3 Further workup of PA cases, radiology, and localization

See **Figure 2** for the study protocol concerning the diagnosed cases of PA. CT of adrenals was performed without contrast. Exclusion of cortisol and catecholamine overproduction was assured with regular biochemical screening. Patients not objecting to eventual surgical treatment (*n* = 45) were referred to Uppsala University Hospital for AVS, which was performed without further adjustment of ongoing medication (MRA already being paused in all patients referred for AVS).

In 52 of the 53 cases with PA, dexamethasone suppression test (DST) was carried out when PA was diagnosed, before proceeding to eventual AVS or PET. In eight patients, DST resulted in serum cortisol slightly above the upper reference level of 50 nmol/L. The highest value was 71 nmol/L (that patient had normal 24-h urinary cortisol). After thorough evaluation, none of these patients was considered to have mild autonomous cortisol secretion, and the cortisol values under AVS did not affect the interpretation of the results.

AVS was performed consecutively, without Synacthen stimulation, and was deemed successful if selectivity index (SI) on each side (plasma cortisol in the respective adrenal vein divided by plasma cortisol in the vena cava inferior) was ≥2.0. Aldosterone production was defined as dominant on one side if the lateralization index (LI, the greater ratio of plasma aldosterone to plasma cortisol in one adrenal vein divided by the smaller plasma aldosterone to plasma cortisol ratio in the other adrenal vein) was ≥4.0. If LI was <4, then the PA was defined as bilateral, except in one case where the clinical decision was taken to consider an LI of 3.2 as a sign of lateralization, and the patient later underwent surgery.

Adrenocortical PET/CT was performed in 11 patients: in 5 patients where AVS was (sometimes repeatedly) unsuccessful, in 5 patients to confirm the result of successful AVS, and in 1 patient without prior AVS. Of these 11 PET/CT procedures, 9 were performed with 11C-metomidate (MTO), which has been used at Uppsala University Hospital for approximately 20 years (34), while the 2 most recent PET/CT procedures were performed with the newly adapted and somewhat more specific tracer para-chloro-2-[^18^F]fluoroethyletomidate (18F-CETO) (35).

The ratio allowing the diagnosis of lateralized PA based on MTO- or CETO-PET/CT was 1.25 between the (higher) standardized uptake value (SUV) in one adrenal divided by the (lower) SUV in the other adrenal. The ratio of 1.25 was chosen after initial studies on MTO-PET/CT performed at the Uppsala University Hospital and Cambridge University (36), which, in retrospect, also served as a basis for the later published MATCH-study (21). On clinical and pragmatic grounds concerning non-inferior sensitivity and specificity of CETO compared to MTO, the same ratio of 1.25 was applied to cases subjected to CETO-PET/CT. However, the optimal ratio for 18F-CETO-PET is under ongoing investigation.

### 2.3 Treatment options

The patients with lateralized aldosterone production were offered laparoscopic unilateral adrenalectomy. The patients not wishing or not suitable for surgical management and those with bilateral PA by AVS or by adrenocortical PET/CT were started on slowly and progressively optimized doses of MRA (eplerenone or spironolactone) under repetitive control of blood pressure, plasma creatinine, sodium, and potassium. The MRA doses were titrated with the intention of having potassium within the higher normal range and DRC (taken at least 2 months after MRA start) no longer suppressed (at least above 8 mIU/L). Other antihypertensive preparations (both in surgically and in medically treated patients with PA) were adjusted to achieve as normalized blood pressure as possible. Clinical follow-up after adrenalectomy or after MRA initiation continued within the study for at least 6–12 months.

### 2.4 Histopathology

Operated specimens underwent histopathologic examination at the Clinical Pathology Department at Uppsala University Hospital using a clinically validated protocol. Both hematoxylin–eosin staining and immunohistochemistry for CYP11B2 were carried out.

### 2.5 Ethics statement

The study was approved by the Swedish Ethical Review Authority and registered on ClinicalTrials.gov (NCT03105531). Data protection laws were adhered to.

### 2.6 Clinical, diagnostic, and laboratory sites

The screening, diagnostic workup, and medical and surgical treatment of diagnosed PA cases were performed at Karlstad Central Hospital. The screening tests were mostly taken by the research nurse. A number of patients (especially during the COVID pandemic) took the screening ARR through the laboratory service of their respective primary care facilities, with the results later analyzed by the research physician. All of the patients who needed to adjust medication within the study did so in direct clinical contact with the research physician (within the Surgical Department of the Karlstad Central Hospital).

Saline suppression testing was done in part by the specialist nurse at the Outpatient Endocrinology Department of Karlstad Central Hospital, and in part by the research nurse at the Surgical Department of the Karlstad Central Hospital.

Aldosterone and renin analyses, AVS, PET/CT, and postoperative histopathology were conducted at Uppsala University Hospital. PAC and DRC were measured by routine clinical analysis (Chemiluminescence Immunoassay, LIAISON Analyzer, DiaSorin Inc., Saluggia, Italy). According to the manufacturer, the LIAISON^®^ Aldosterone analysis had a limit of quantitation of 40.2 pmol/L and reliably measured aldosterone concentration up to 2,770 pmol/L. The functional sensitivity of the LIAISON^®^ Renin analysis was 1.6 mIU/L, with the reliably detected concentration up to 500 mIU/L. There was linearity in measurement of these substances within the named ranges.

### 2.7 Statistical analysis

Variables were expressed as mean (standard deviation, SD) where parametric methods were employed, or as median [interquartile range (IQR)] if nonparametric methods were used. Categorical variables were expressed as absolute numbers and percentages. Differences between groups with nonparametric testing were analyzed by the Mann–Whitney *U* test or the Wilcoxon signed-rank test. When appropriate, the Student’s paired *t*-test or (if equality of variances was not obviously present) the Welch *t*-test was used. Categorical data were assessed by chi-square or Fisher’s exact test. All the statistical tests used were two-sided. *p* 0.05 was considered significant. IBM SPSS Statistics version 26 software was used for statistical analysis.

## 3 Results

Baseline clinical characteristics of the *total* study population including current medication are presented in **Tables 2** and **3**. Screening of 1,181 persons resulted in 53 found cases of PA, amounting to a prevalence of 4.5%. See **Tables 2** and **4** concerning the clinical characteristics and medication for the groups of PA and primary (essential) hypertension HT.

**Table 2:** Demographic and clinical characteristics of the groups within the study [values are expressed as median (interquartile range, IQR) or *n* (%)].

		Total study population *n* = 1,181	Primary aldosteronism (PA) *n* = 53	Primary hypertension (HT) *n* = 1,128	Difference between PA and HT, *p* ^a^
Male		580 (49%)	27 (51%)	553 (49%)	0.785
Female		601 (51%)	26 (49%)	575 (51%)	
Age (years)		58 (53–62)	57 (53–61)	58 (53–62)	0.30
BMI		28.4 (25.6–31.6)	29.4 (25.9–33.1)	28.3 (25.6–31.5)	0.12
Blood pressure at screening	Systolic	138 (15)	145 (16)	138 (14)	0.005^*^ ^c^
	Diastolic	87 (9)	91 (10)	87 (9)	0.003^*^ ^c^
Plasma creatinine, μmol/L		69 (61–80)	73 (63–80)	69 (61–80)	0.34
eGFR, mL/min^b^		83 (76–90)	82 (75–90)	83 (76–90)	0.79
Plasma sodium [mean (SD)]		139.9 (2.37)	140.6 (1.83)	139.9 (2.39)	0.015^*^ ^c^
Plasma potassium [mean (SD)]		3.9 (0.29)	3.7 (0.32)	4.0 (0.28)	<0.001^*^ ^c^
Smoking now		84 (7%)	3 (6%)	81 (7%)	0.674
Former smoking		537 (46%)	21 (40%)	516 (46%)	0.382
Use of chewing tobacco (Swedish “snus”)		175 (15%)	7 (13%)	168 (15%)	0.922
Former use of chewing tobacco (“snus”)		317 (27%)	12 (23%)	305 (27%)	0.752
Regular consumption of licorice		120 (10%)	3 (6%)	117 (10%)	0.846
Age (years) when hypertension was discovered		50 (40–55)	44.5 (38–53)	50 (40–55)	0.013^*^
Angina pectoris/myocardial infarction/percutaneous coronary intervention in the anamnesis		90 (7.6%)	7 (13%)	83 (7.4%)	0.467
Ischemic cerebrovascular lesion/transitory ischemic attack in the anamnesis		53 (4.7%)	0	53 (4.7%)	0.438
Hemorrhagic stroke in the anamnesis		7 (0.6%)	0	7 (0.6%)	0.828
Peripheral atherosclerosis		9 (0.8%)	0	9 (0.8%)	0.789
Chronic heart failure		5 (0.4%)	0	5 (0.4%)	0.868
Diabetes mellitus or glucose intolerance		188 (15.9%)	8 (15.1%)	180 (16.0%)	0.963
Chronic obstructive lung disease		19 (1.6%)	0	19 (1.7%)	0.620
Chronic renal failure		6 (0.5%)	1 (2%)	5 (0.4%)	0.344
Atrial fibrillation/flutter/other significant acquired chronic arrythmia		50 (4.2%)	2 (4%)	48 (4.3%)	0.962
Hyperlipidemia^d^		370 (31.3%)	15 (28%)	355 (31.5%)	0.866
History of pulmonary embolism		21 (1.8%)	0	21 (1.9%)	0.591
Osteoporosis		19 (1.6%)	0	19 (1.7%)	0.576
Sleep apnea syndrome		137 (11.6%)	7 (13%)	130 (11.5%)	0.973

a *p* according to Mann–Whitney *U* test or, respectively, chi-square or Fisher’s exact test.

b eGFR (mL/min), estimated glomerular filtration rate, MDRD formula (Modification of Diet in Renal Disease Study Group).

c *p* according to Welch’s *t*-test.

d Hyperlipidemia was defined as chronic use of statins of other prescribed lipid-lowering medication.

* and data in bold denote statistically significant difference.

**Table 3:** Current medication for the total study population at the time of screening [*n* = 1,181 (%)].

Number of antihypertensive medicines	0	*n* = 57 (4.8%)
	1	*n* = 501 (42.4%)
	2	*n* = 382 (32.3%)
	3	*n* = 192 (16.3%)
	4	*n* = 42 (3.6%)
	5	*n* = 7 (0.6%)
Antihypertensive medicines		
Angiotensin receptor blockers		50.6%
Dihydropyridine calcium antagonists		40.9%
Angiotensin-converting enzyme inhibitors		27%
Thiazide diuretics		25.1%
Beta blockers		22.4%
Alfa blockers		2%
Mineralocorticoid receptor antagonists		1.9%
Loop diuretics		1.4%
Potassium-sparing diuretics (Amiloride)		1%
Central calcium antagonists (Verapamil)		0.4%
Imidazoline receptor antagonists		0%
Renin inhibitor (Aliskiren)		0%
Other relevant medicines		
Combined estrogen–progesterone preparations		1.4%
Estrogen preparations		6.1%
Selective serotonin reuptake inhibitors		7.6%
Non-steroid anti-inflammatory inhibitor drugs		13.5%

**Table 4:** Number of groups of antihypertensive medicines in the study groups at the time of screening.

		Total study population, *n* = 1,181	Primary aldosteronism (PA), *n* = 53	Primary hypertension (HT), *n* = 1,128	Difference between PA and HT
Number of groups of antihypertensive medicines	0	57 (4.8%)	4 (7.5%)	53 (4.7%)	Fisher’s exact test *p* < 0.001 *
	1	501 (42.4%)	16 (30.2%)	485 (43%)	
	2	382 (32.3%)	9 (17%)	373 (33%)	
	3	192 (16.3%)	17 (32.1%)	175 (15.5%)	
	4	42 (3.6%)	5 (9.4%)	37 (3.3%)	
	5	7 (0.6%)	2 (3.8%)	5 (0.4%)	
Number of groups of antihypertensive medicines	Mean (SD)	1.7 (0.95)	2.2 (1.28)	1.7 (0.93)	Mann–Whitney *U* test *p* = 0.006 *
	Median (IQR)	2 (1-2)	2 (1-3)	2 (1-2)	
Mean rank for Mann–Whitney *U* test			798	585	

*Statistically significant.

Subjects not diagnosed with PA were clinically assessed on an individual basis for other possible causes of secondary hypertension. No cases that would benefit from other relevant specific treatment were found, and these patients continued to be taken care of by their primary care physician with the diagnosis of HT.

Most baseline clinical characteristics were comparable between the groups of HT and PA. However, the patients with PA had significantly higher blood pressure, which also was discovered at an earlier age than in the HT group. The PA group had initially slightly but significantly higher sodium and lower potassium in plasma (**Table 2**). The number of initially used antihypertensive medicines was slightly but significantly higher for patients with PA than for those with HT (**Table 4**).

**Figure 3:**
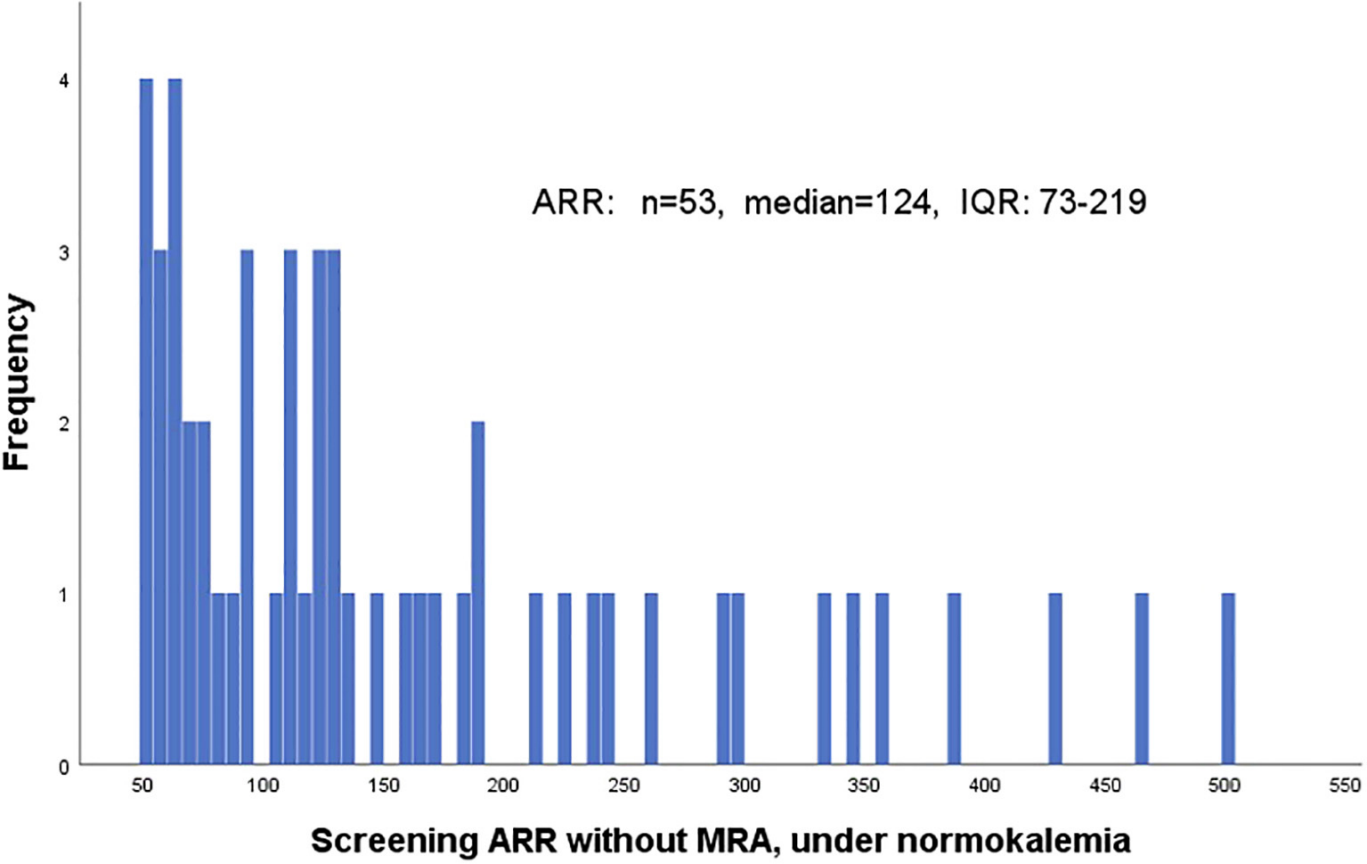
Frequency distribution of aldosterone–renin ratio among the diagnosed cases of primary aldosteronism—at the moment of the screening. ARR, aldosterone–renin ratio (pmol/mIU); IQR, interquartile range; MRA, mineralocorticoid receptor antagonists.

The ARR (without MRA and under normokalemia) in cases diagnosed with PA ranged between 50 and 500 pmol/mIU (**Figure 3**). In total, 20 patients (38%) had ARR 50–100 pmol/mIU and 13 (25%) had ARR 100–150 pmol/mIU. Considering the initial clinical parameters and the medical history of each patient with PA found in the study, only approximately 60% of them would have been included in the risk groups to be screened according to the latest guidelines (20).

Of all excluded patients, 15 were excluded directly after inclusion due to perceived high risk of diagnostic workup with regard to comorbidities (*n* = 10, 5 of whom with heart failure), risk of MRA discontinuation (*n* = 3), or difficult vein access (*n* = 1). One patient who was followed by both a primary care and a specialized hypertension clinic was excluded.

In patients who underwent ARR#2 due to hypokalemia and not due to initial use of MRA (*n* = 25), potassium at the time of ARR#1 [median 3.3 (IQR 3.2–3.4)] was lower than at ARR#2 [3.8 (3.6–3.9)], Wilcoxon signed-rank test *p* < 0.001. In spite of that, the difference between ARR#1 [median 6.3 (IQR 1.8–16.3)] and ARR#2 [10.4 (3.1–28.9)] was not significant (Wilcoxon signed-rank test *p* = 0.143).

Eight patients of those initially on MRA (*n* = 22) were diagnosed with PA. In six of these, renin was below normal at ARR#1, and in another two patients, it was in the middle of the reference range. Remarkably, all of these eight patients had ARR#1 > 50, in spite of the ongoing use of MRA.

### 3.1 Confirmation testing

Therapy adjustment between rSST#1 and rSST#2 was well-tolerated. One patient was excluded due to anticipated high risk from therapy adjustment, and another patient was excluded due to worsening of blood pressure and heart failure symptoms concomitant with medication changes before rSST#2.

Of all patients lost to follow-up, five discontinued participation in the study during the process of therapy adjustment before rSST#2, without giving the study personnel any particular reason. None of the 40 patients who completed rSST#2 displayed any related complications.

Final aldosterone levels after the diagnostic SST in the group with lateralized PA were significantly higher than those in the group with bilateral PA, 320 (270–464) pmol/L vs. 246 (203–300) pmol/L, Mann–Whitney *U* test *p* = 0.009.

### 3.2 Aldosterone, renin, and ARR at different stages

Aldosterone, renin, and ARR were compared during different stages of the diagnostic process. ARR taken just at the start of rSST#1 (*n* = 113; “first ARR under rSST#1”) was compared with the last ARR taken before that (“last ARR before rSST#1”). Paired *t*-test was possible and showed a significantly lower level of the “first ARR under rSST#1” in comparison with the “last ARR before rSST#1.” Mean difference (95% confidence interval, CI) was 28 pmol/mIU (CI 15-41), *p* < 0.001 (because 14 outliers did not significantly affect the results after their exclusion, they were left in place for the final calculation of the paired *t*-test).

Comparison of PAC-4h after rSST#1 and after SST#2 showed significantly higher aldosterone after rSST#2, after excluding one outlier of the data from 40 patients. Mean difference was 28 pmol/L (95%CI 12-44), *p*=0.001.

The ARR at the initiation of rSST#1 was significantly lower (*p* = 0.048) than that at the initiation of rSST#2 (*n* = 40), after excluding four outliers allowing a paired *t*-test. The mean difference between ARR#1 and ARR#2 was −22.5 pmol/mIU (−0.3 to −44.8).

### 3.3 Lateralization of PA

Of 53 patients with PA, 44 agreed to and underwent AVS (**Figure 2**), permitting bilateral PA to be diagnosed in 28 cases and lateralized PA in 11. In two of these patients, AVS was interpretable only when repeated the second time. AVS failed in five cases (and in two of them, twice) due to technical difficulties during catheterization and low SI. These five patients were subjected to PET/CT (showing three symmetrically bilateral and two lateralizing forms of PA). Another patient underwent PET/CT without prior AVS, with the uptake ratio signifying lateralized disease. Thus, in total, 31% (14 out of 45) of all who underwent AVS or PET/CT demonstrated lateralization. There were no complications related to AVS or PET.

### 3.4 Operated patients

Of the 14 lateralized cases, 11 have been operated with unilateral laparoscopic adrenalectomy (see **Figure 2**). Postoperative results and histopathology were assessed using the PASO (37) and HISTALDO (30) criteria. Four of the operated patients had an APA, five patients had multiple APM, while in one patient, only one APM was described. In one case, there were multiple APNs including one dominant APN.

Three of the patients with unilateral PA have not been operated. Thus, at least 4 out of 11 (36%) operated, but possibly up to 7 out of 14 lateralized (50%), and 4–7 out of 45 subtype-classified patients with PA (9%–16%) may have had APA.

**Figure 4:**
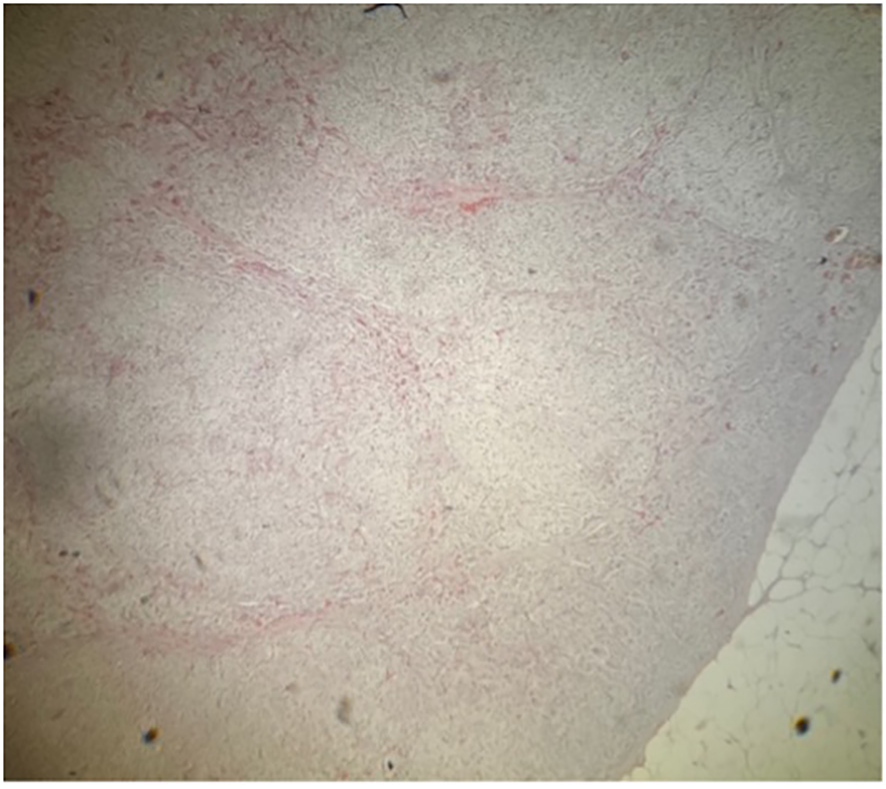
Histopathology of specimen from the adrenal gland in a patient with PA. Part of CYP11B2-positive nodule A, hematoxylin–eosin staining. Magnification ×40.

**Figure 5:**
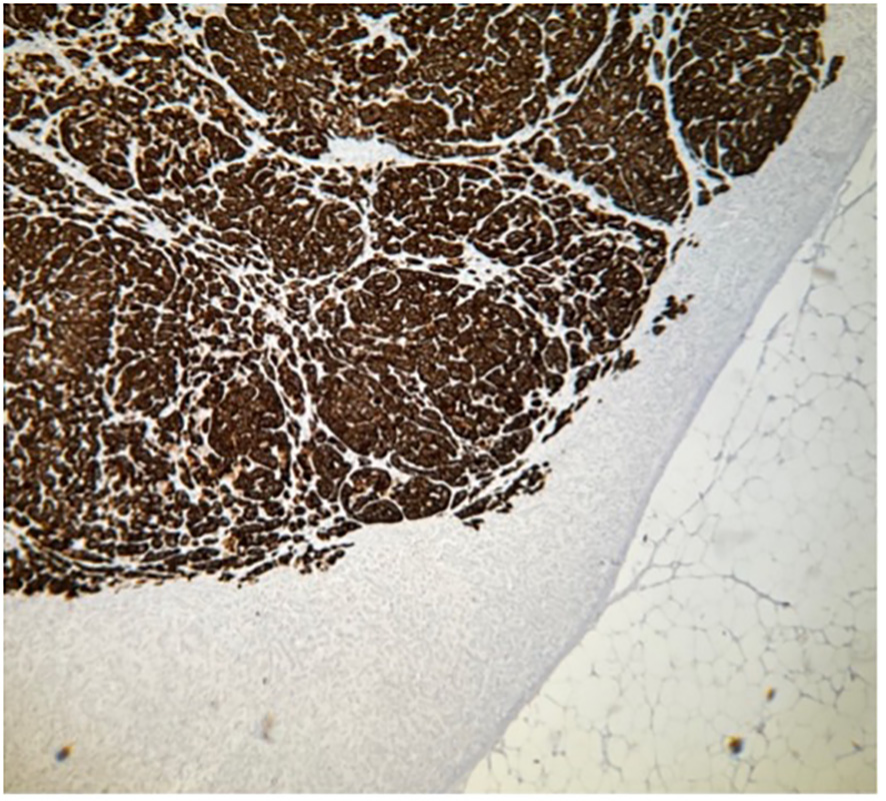
Histopathology of specimen from the adrenal gland in a patient with PA. Part of CYP11B2-positive nodule A, immunohistochemistry demonstrating expression of aldosterone synthase, CYP11B2. Magnification ×40.

**Figure 6:**
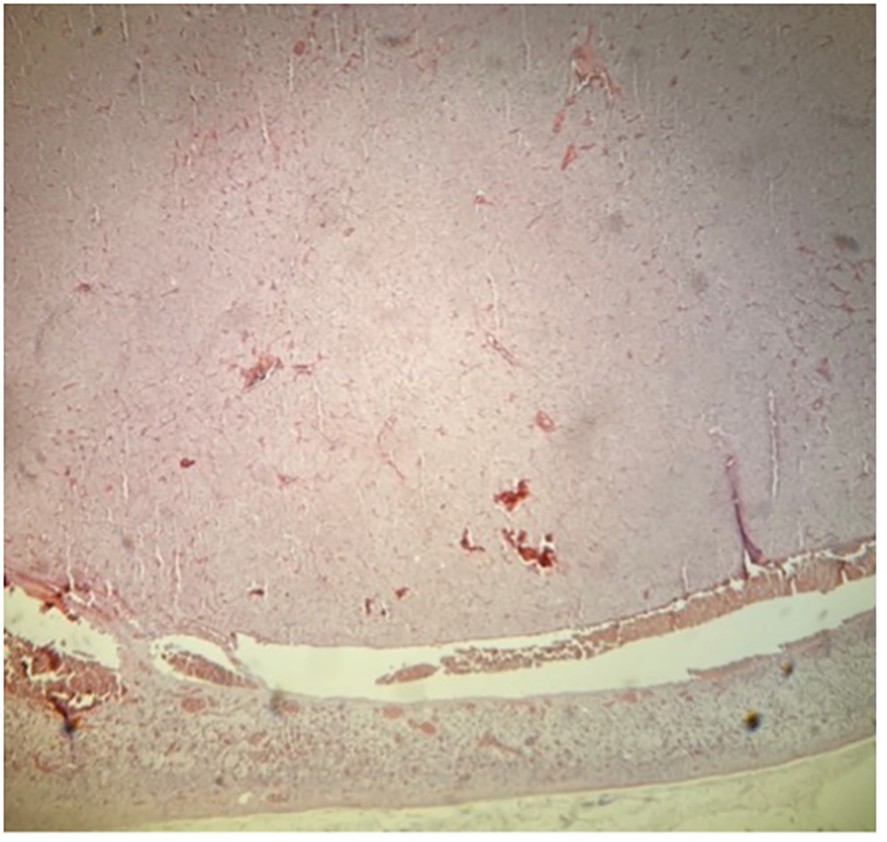
Histopathology of specimen from the adrenal gland in a patient with PA. Part of CYP11B2-negative nodule B, hematoxylin–eosin staining. Magnification ×40.

**Figure 7:**
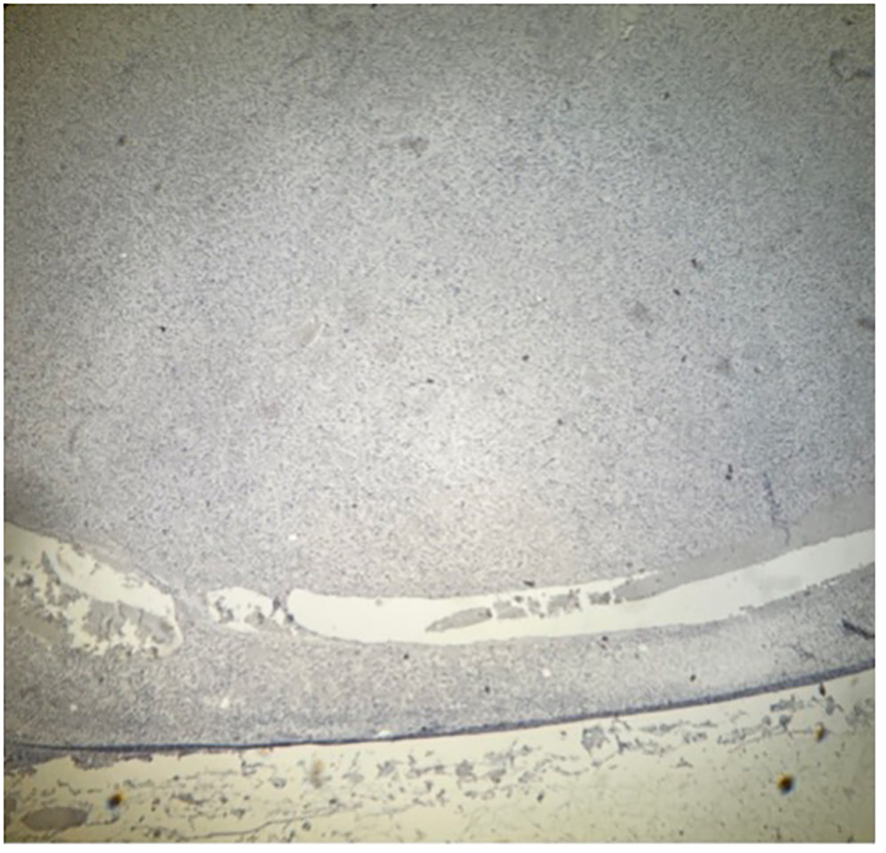
Histopathology of specimen from the adrenal gland in a patient with PA. Part of CYP11B2-negative nodule A, immunohistochemistry demonstrating absent expression of aldosterone synthase, CYP11B2. Magnification ×40.

Consequently, 7–10 out of 14 lateralized cases had some form of nodular disease or true hyperplasia (50%–71%), which supports previous data (31, 38). Assuming that the 31 patients who did not lateralize also had some form of non-APA disease, the total number of patients with non-APA disease approaches 38–41 out of 45 subtype-classified patients with PA (84%–91%). See **Figures 4**–**7** for histological examples of CYP11B2-ICH and its value.

The four cases of adenoma all had complete biochemical success of surgery. In the seven cases of nodular pathology (including the case with a dominant APN), four demonstrated complete postoperative biochemical success; in the other three cases with partial biochemical success (all with APMs), low doses of MRA had to be initiated postoperatively. Complete clinical success (where the patient discontinued all antihypertensive medication postoperatively) was only seen in one case with APA, a 56-year-old female patient with a 25-year history of hypertension.

Of all operated patients, there was one case of postoperative pulmonary embolism in spite of pharmacological anti-thrombotic prophylaxis. Temporary cortisol substitution was needed in two cases—in spite of adequately low preoperative dexamethasone suppressed cortisol (24 and 40 nmol/L, respectively). No other postoperative complications occurred.

### 3.5 Conservatively treated PA

A total of 42 patients with PA were started on MRA, with the dosage gradually augmented with the intention to reach adequate elevation of plasma renin while keeping serum potassium within the higher normal reference range. These 42 patients were composed of 31 patients with bilateral forms (28 at AVS, 2 at MTO-PET, and 1 at CETO-PET), 8 patients who refrained from AVS, and 3 unilateral patients (2 at AVS and 1 at PET) who finally had to be treated conservatively. Within a median of 13 months (IQR 12–17), 40 of these patients (95.2%) were considered to have reached the optimal possible MRA therapy. The three lateralized patients with PA where conservative treatment was chosen consisted of (i) one patient with a gigantic adrenal cyst and local pressure symptoms demanding adrenalectomy on the opposite side to the aldosterone-overproducing adrenal; (ii) one patient with morbid obesity; and (iii) one patient who declined surgery and already was treated successfully with MRA. The three cases with partial biochemical success postoperatively (all with non-APA forms of aldosterone overproduction) were started on low doses of MRA and responded with an adequate rise in renin.

An adequate rise of DRC was achieved at the latest follow-up in 51 out of 53 patients who completed the follow-up, and was >8.0 mIU/L in 44 of these patients (86%). A DRC value of 8 mIU/L corresponds to a plasma renin activity (PRA) value of 1 μg/L per hour—shown to be the minimal protective plasma renin value for conservatively treated patients (18, 19). DRC below 8 mIU/L was still present in two patients with complete biochemical success after adrenalectomy as shown by low ARR (23 and 17 pmol/mIU, respectively) and low-normal PAC (122 and 75 pmol/L, respectively). In addition, in five of the conservatively treated patients, final DRC was below 8 mIU/L. In two of them, this was deemed due to the concurrent administration of beta blockers or NSAIDs that could not be paused; one patient with totally normalized blood pressure on a given dose of MRA wished not to raise the dosage further in spite of low final renin; one patient, treated only with MRA, could not further raise their dosage due to hypotension; and one patient had reported side effects (fatigue and headache) of consecutively tested spironolactone, eplerenone, and amiloride and was thus left on a calcium blocker.

Overall, DRC increased from 4.2 (IQR 2.0–5.7) mIU/L (*n* = 51), measured just before the SST, to 20 (IQR 9.4–41) mIU/L at the latest follow-up (Wilcoxon signed-rank test, *p* < 0.001).

### 3.6 Treatment outcome

Of 53 PA cases, 23 had potassium supplementation before ARR screening, and none at follow-up (Fisher’s exact test *p* < 0.0001). Potassium levels were significantly higher after adequate treatment [mean 4.26 (SD 0.28) mmol/L] than before the screening [3.73 (0.32)]; *n* = 46, *p* < 0.001.

The mean number of groups of antihypertensive medication at inclusion was 2.2 (median 2, IQR 1–3), while at the latest follow-up, the mean was 2.6 (median 2, IQR 2–4), Wilcoxon signed-rank test *p* = 0.020 (*n* = 51). The aim during the final adjustment of medication was to achieve normal blood pressure if possible, which may well have caused the number of blood pressure medications to rise for the study patients. A positive effect of study-related treatment on blood pressure was noted (see **Table 5**).

**Table 5:** Specific treatment of primary aldosteronism: effect on blood pressure (*n* = 51) evaluated after follow-up of median 12 months (IQR 12–17, range 6–33).

	BP^a^ before specific PA treatment (on antihypertensive drugs before diagnosis of PA)		BP after specific PA treatment		Paired *t*-test
	Systolic	Diastolic	Systolic	Diastolic	
Mean (SD)	145 (16)	91 (10)	126 (9)	79 (5)	*p* < 0.001 *

a BP, blood pressure.

*Statistically significant.

## 4 Discussion

This study demonstrates that PA is present in 4.5% of a cohort of unselected primary care patients with hypertension, bearing in mind that the screened individuals had predominantly moderate hypertension and were, in most cases, screened during ongoing medication (which was one of the aims of the study). The majority of PA cases found were mild. Adjustment of medication, when necessary, could be carried out safely, but was time-consuming. Several patients with a higher probability of PA, such as those with higher stages of heart failure, could not be subjected to the complete diagnostic process, which underscores the need for optimized diagnostic methods. Moreover, we did not account for any earlier diagnosed cases of PA already followed by a specialist clinic. Several authors underline the uncertainty in the estimation of individual ARR (9, 39–41), which allows possible underdiagnosis of PA when ARR is used for screening, and the search continues for alternative and better biochemical screening for PA (42). Some advocate the use of urinary aldosterone excretion to more reliably find the true prevalence of PA (8, 15, 43). As an illustration for the inherent limitations of existing screening tests, a higher prevalence of PA has been found in studies avoiding the screening test and directly using confirmatory procedures (5). Therefore, the actual prevalence of PA among the total hypertensive population in Sweden is most likely above 4.5%, while this number may well be accurate in the cohort of individuals with milder hypertension followed within primary care in Sweden.

One of the study aims was to investigate whether screening for PA with ARR under ongoing antihypertensive medication, but without MRA (thus simplifying interpretation of the Endocrine Society guidelines), was possible—a hypothesis that our results support. There are some, but not many, studies designed to answer a similar question (44–46). The patients in our project were selected based on the presence of hypertension, and not upon its severity, related symptoms, or the patient’s age when hypertension or complications developed, which represents one of the strengths of our study. The guidelines (20) recommend screening for patients in certain risk groups. In our material with mostly mild cases, approximately 40% of patients with diagnosed PA did not belong to such risk groups. Indeed, several research teams recommend screening of all patients with hypertension at least once (7, 15, 47–49).

There is a continuing debate whether screening and confirmation of PA should include preliminary discontinuation of medication known to possibly affect renin and aldosterone (15, 39, 50–54). Even the guidelines are equivocal on the subject (20). The majority of such preparations (besides MRA) can lower aldosterone and (besides betablockers) elevate renin. As each SST implied measurement of “initial” ARR and PAC-4h, we could demonstrate that these values were significantly higher during rSST#2 compared with rSST#1. Thus, our results support that maximal acceptable discontinuation of antihypertensive drugs may contribute to successful evaluation of the final diagnostic procedures.

As mentioned above, at the time of the study’s protocol planning, the recumbent SST was a state-of-the-art method for this confirmatory test. Since then, the approach has developed, and it is currently widely accepted that the blood tests during screening and confirmation procedures such as SST should be taken with the patient seated and not recumbent, which further elevates the sensitivity of these procedures (55, 56). The difference between the last ARR before rSST#1 (while sitting) and the initial ARR at rSST#1 (after some minutes lying down) serves as an illustration of this finding.

The role of normokalemia at the time of hormonal evaluation should not be seen as negated by our results. Rather, the results may be seen as indicating only a minor effect of *mild* hypokalemia on ARR.

The majority of scientific publications to date adhere to the longstanding tradition of dividing the anatomical subtypes of PA into “unilateral APA,” representing 30%–40% of cases; “bilateral hyperplasia,” representing 60%–70% of cases; and other seldom occurring forms of PA, sometimes mentioned as “unilateral hyperplasia.” There is, however, growing evidence of the existence of lateralized PA caused by APNs or APMs rather than an APA, which also can be successfully treated with adrenalectomy (30, 31, 38, 57, 58). Unilateral diffuse aldosterone-overproducing hyperplasia is rare (31, 32, 38). Iacobone et al. have noted that up to 74% of AVS-defined unilateral PA cases were represented by non-adenoma lesions (even if IHC was not performed, 38). Our results support this finding, with APA being present in 29%–50% of the patients with lateralized PA. Iacobone et al. also noted that unilateral adrenalectomy in these cases was as highly effective at 3 years’ follow-up as in those with a clear adenoma in the postoperative specimen (38).

Surgical treatment of unilateral forms of PA is associated with a substantial long-term (mainly cardiovascular) risk reduction (16). In contrast, patients with PA offered specific and continued pharmacological treatment (especially in those cases when it is not sufficient enough to counteract renin suppression) still suffer from significantly greater secondary organ damage and increased mortality (17, 18, 59, 60). Thus, identification of lateralizing cases is important, in order to recommend adrenalectomy in these patients.

Lateralized PA is generally characterized by more pronounced aldosterone overproduction than non-lateralized forms, which may facilitate limiting the number of patients for localizing studies as those are essentially needed just for the potentially operable cases (61, 62). This paradigm is even illustrated in our material where final aldosterone level after diagnostic SST was significantly higher among the lateralized cases compared to the bilateral cases. In contrast, in patients with mild forms of PA, which are predominantly bilateral, as also noted in our cohort, localizing studies could be omitted (10, 63). Conservative treatment in such individuals, controlled by rising renin, may constitute a sound pragmatic alternative. Further development and validation of non-invasive adrenal cortex-specific PET/CT may also simplify the workup. Practically, the results of specific treatment of the PA cases discovered in the current cohort reveal that it is clinically safe and possible to apply the principles of such treatment.

In spite of recent years’ attention to PA, the rate of screening, detection, and adequate management of the disease is still very low (43, 64–66). A recent Swedish register-based study of the incidence of PA has documented progressive growth of its diagnostic discovery within the population of a large region during the last years—but still to the degree that corresponds to a far lower number of diagnosed patients with PA than expected (67). Awareness of healthcare professionals—especially primary care practitioners—would be essential in the process of diagnosing the disorder and the risks it implies (13, 66).

The role of screening for PA in hypertensive populations is not merely to improve control of blood pressure. It is also important to adequately reduce the negative effects of high non-physiologic aldosterone levels. We recommend liberal screening by ARR in all hypertensive adults.

## 5 Limitations

The prevalence of PA found in our study may reasonably be seen through the prism of the factors that could lead to missed cases during screening. Some missed cases might exist among those who never responded to the invitation to participate in the study.

Screening under ongoing antihypertensive treatment (without MRA, but often including preparations potentially affecting RAAS) may have resulted in some number of false-negative individuals. Of course, one has to be careful in the interpretation of biochemical PA screening data obtained under ongoing medication. To reduce this risk, we would have needed to safely adjust the medications in *all* patients before screening, which is cumbersome, excessively time-consuming and labor-intensive, and requires a high degree of motivation from the patients to be able to succeed. These efforts were made among those who underwent rSST#2, thus only a subcohort. Our understanding is that it would not be possible to accomplish that kind of task within available resources for this study, and it was not coherent with our intentions to simplify the diagnostic protocol.

During rSST#1, both recumbency and ongoing medical treatment could have led to false-negative results. We used recumbent SST in the screening, but more recently, sitting SST has been proven superior. Traditionally, the older scientific publications and textbooks presented an axioma that abnormal autonomous aldosterone production should necessarily be unrelated to angiotensin-2 stimulation. The “rule” was shown to be erroneous by a number of studies (55, 56, 68) that illustrate that the majority of cases with PA are sensitive to that stimulation. Furthermore, some forms of dysregulation imply secretion of excessive amounts of aldosterone under physiologic stimulation—such as by adrenocorticotropic hormone (ACTH) released during stress and, to some extent, also by renin (69). Aldosterone hypersecretion due to stimulation by ACTH has been shown to be more pronounced for unilateral forms of PA (as opposed to bilateral PA) and could be even used to differentiate unilateral from bilateral PA (70).

Another limitation of the study is the modest number of patients found and treated for PA. The fact that they coherently originate from an unselected primary care hypertensive population may, on the other hand, be seen as a strong advantage, thus detailing the profile of PA that may be the one more typical for the general majority of the cases.

## 6 Conclusion

a. Screening for PA in individuals with hypertension within primary care is safely performed using the Endocrine Society guidelines.
b. PA is common among primary care patients with hypertension and should be actively screened for to avoid premature morbidity and mortality, which is also present in milder forms of PA.
c. We advocate this screening for all patients with hypertension, at least under a certain pragmatically defined age.
d. Screening may be performed with plasma ARR taken while sitting, and during ongoing antihypertensive medication, but without MRA.
e. A significant number of patients diagnosed with PA in this screening had ARR just above 50 pmol/mIU, and this cutoff may be used in clinical practice.
f. Discontinuation of medicines that affect ARR may unmask pathologic ARR and raise aldosterone in suppression testing. Therefore, this should be clinically considered if suspicion of drug-dependent false-negative outcome of biochemical hormonal testing exists.
g. Mild hypokalemia affects the results of such testing to a lesser extent. Both screening and further workup are in need of more straightforward and sensitive diagnostic tools for PA.
h. It is important to suggest that a patient who has undergone negative screening for PA by ARR should not automatically for life be considered as having primary hypertension. Re-screening should be performed if clinically motivated.
i. Mild PA is less often caused by APA than more clinically florid forms of PA. High prevalence of nodal forms and relatively low prevalence of APA (according to HISTALDO criteria) may be features of the lateralizing PA cases found in a setting of liberal screening within primary care patients with hypertension.
j. We also describe the use of adrenocortical PET/CT among the localizing studies and foresee an increased role for this non-invasive method in the future.
k. Clinical work within the study (including all the aspects of treatment) was carried out in a regional hospital (with AVS and PET done at the University Hospital). It is not improbable that the scope of PA diagnosis in the adult population one day reaches quite considerable volumes. In that case, the study may serve as an example of feasibility of decentralization of practical treatment for PA.

## Data Availability

The raw data supporting the conclusions of this article will be made available by the authors, without undue reservation.
